# Impact of Decision Aids for Shared Decision-Making for Patients with Early Stage Breast Cancer: A Systematic Review

**DOI:** 10.3390/curroncol33090518

**Published:** 2026-08-30

**Authors:** Serena Sdinami, Lorenzo Conti, Benedetta Capetti, Valeria Sebri, Paola Zagami, Roberto Grasso, Carmen Criscitiello, Giuseppe Curigliano, Gabriella Pravettoni

**Affiliations:** 1 Applied Research Division for Cognitive and Psychological Science, IEO, European Institute of Oncology IRCCS, Via Ripamonti, 435, 20141 Milan, Italy; 2 Department of Oncology and Hemato-Oncology, University of Milan, 20123 Milan, Italy; 3 Division for New Drugs and Early Drugs Development for Innovative Therapies, IEO, European Institute of Oncology IRCCS, 20141 Milan, Italy

**Keywords:** early stage breast cancer, patient decision aid, shared decision-making, decision quality, health literacy, implementation

## Abstract

Objectives: To systematically review the evidence on the effectiveness of patient decision aids (PDAs) for women with early stage breast cancer, focusing on decision-making outcomes, psychological variables, and quality of life. Methods: A systematic review was conducted in accordance with PRISMA guidelines. PUBMED, EMBASE and SCOPUS databases were searched to identify quantitative, qualitative and mixed-methods studies evaluating PDAs designed to support treatment decision-making in women with early stage breast cancer. Eligible studies included randomized controlled trials, quantitative, qualitative and mixed-methods studies. Outcomes of interest included knowledge, decisional conflict, Shared Decision-Making (SDM), anxiety, depression, and quality of life. Special focus has been given to lower Socio-Economic Status (SES) and lower literacy populations. Results: Twenty-six studies (5174 participants) met inclusion criteria. PDAs were associated with reduced decisional conflict (6 of 7 studies), improved satisfaction with the decision or decision-making process (4 studies), and enhanced knowledge (4 of 8 studies showing statistically significant improvement). No consistent adverse effects on anxiety or depression were observed. Ten studies focused on lower SES or low-literacy populations, highlighting the effectiveness of pictorial and simplified PDAs in improving knowledge, SDM, and reducing decisional regret. Qualitative findings indicated high acceptability but identified barriers related to timing and institutional implementation. Conclusions: PDAs for women with early stage breast cancer enhance knowledge and support informed, value-congruent treatment decisions. However, evidence regarding their impact on psychological well-being and long-term quality of life remains inconclusive. Further research should also address institutional implementation policies.

## Simple Summary

Women with early stage breast cancer often need to choose between several treatment options, and these decisions can be difficult and stressful. Patient Decision Aids are tools designed to support these choices by providing clear, evidence-based information and helping patients think about what matters most to them. In this systematic review, we analyzed 26 studies involving 5174 women with early stage breast cancer to evaluate whether these aids improve decision-making, knowledge, psychological well-being and quality of life, while also gathering qualitative data regarding the acceptability and feasibility of these tools. We also dedicated a special focus to undeserving or lower health literacy populations. Overall, Patient Decision Aids were associated with better knowledge, less decisional conflict and greater satisfaction with the decision-making process. Some studies also suggested benefits for women with lower health literacy or socioeconomic status, especially when the aids used pictures or simple language. However, effects on anxiety, depression and quality of life were less consistent. These findings suggest that Patient Decision Aids can be useful tools in breast cancer care, but their impact depends on how they are designed and implemented.

## 1. Introduction

Breast cancer (BC) is the most common cancer among women worldwide [1]. Prognosis varies according to disease stage and clinicopathological characteristics, with early diagnosis being associated with improved prognosis and survival [2]. According to the latest edition of the American Joint Committee on Cancer (AJCC) Tumor, Node, Metastasis (TNM) staging system, early stage BC comprises non-metastatic stages 0, I, II, and III, with stage III including locally advanced and, in some cases, inoperable disease [3].

Treatment options for early stage BC include both local and systemic therapies. Local treatments consist of surgery and radiotherapy, whereas systemic therapies include chemotherapy, endocrine therapy, targeted therapies, and immunotherapy [2]. Surgery followed by radiotherapy remains the standard of care for many patients. However, advances in breast-conserving surgery and reconstructive techniques have expanded the range of available surgical options, requiring patients and healthcare professionals to jointly consider prognosis, comorbidities, patient well-being, and individual preferences when selecting the most appropriate treatment. For example, patients may choose between breast-conserving surgery (lumpectomy, performed through either volume displacement or volume replacement techniques) and different types of mastectomy, including modified radical and nipple- or skin-sparing procedures [4].

Systemic treatment decisions are primarily guided by the tumor’s histopathological characteristics and the expression of biomarkers, such as hormone receptors and HER2. However, in situations where evidence is limited or uncertainty regarding outcomes exists, treatment selection increasingly relies on patients’ preferences and values, which may vary substantially across individuals [5,6]. These situations are commonly referred to as preference-sensitive decisions, in which patients’ values play a central role because treatment options often involve trade-offs between potential benefits and risks. To support these complex decisions, the concept of Shared Decision-Making (SDM) has been developed. SDM is defined as an approach in which clinicians and patients share the best available evidence while jointly considering available treatment options, with the ultimate goal of reaching informed decisions that reflect patients’ preferences and values [7].

SDM refers to the active participation of both physicians and patients throughout all phases of the decision-making process, from information exchange to treatment deliberation and the final decision [8]. Previous research has shown that effective communication during clinical encounters, together with physicians spending time educating, counseling, and guiding patients through the available treatment options, is associated with improved clinical outcomes, greater treatment adherence, and higher patient satisfaction [9,10]. To facilitate SDM, Patient Decision Aids (PDAs) have been developed. PDAs are adjuncts to physician–patient discussions during treatment decision-making [11]. More specifically, they are interventions designed to help individuals make informed and deliberate choices among different treatment options by providing evidence-based information tailored to their health condition, while also helping patients clarify their personal values and preferences [11]. PDAs can take various forms, including printed booklets, videos, web-based platforms, and interactive multimedia applications. Their primary purpose is to provide information about the disease, available treatments, and expected outcomes while supporting patients in identifying which aspects of care are most relevant to their individual circumstances.

A growing body of evidence suggests that the use of PDAs among patients with cancer improves knowledge and satisfaction with the decision-making process and, in some cases, may also influence the final treatment choice [12,13,14,15]. Among patients with early stage BC, PDAs implemented before surgery have been shown to positively influence treatment decision processes and final choice of treatment [16]. Moreover, women with BC consistently report preferring an active role in treatment decision-making and value sharing decisions with their physicians [17,18]. Evidence further indicates that patients who actively participate in oncology consultations experience better decision-related outcomes over the long term [19], whereas those who are less involved in treatment decisions are more likely to report psychological distress and depressive symptoms [20].

The objectives of the present review are, therefore, to: (i) identify currently available PDAs developed for women with early stage BC within the SDM framework; (ii) identify the main outcomes and assessment tools used to evaluate the effects of PDAs; (iii) systematically evaluate the effectiveness of PDAs for women with early stage BC; and (iv) evaluate the acceptability, feasibility, and usability of these interventions. Given the documented benefits of PDAs among individuals with lower Socio-Economic Status (SES) or limited health literacy [21], we also aimed to specifically examine PDAs that have been developed or implemented for these underserved populations.

## 2. Methodology

The protocol for this systematic review was prospectively registered in the PROSPERO International Prospective Register of Systematic Reviews database before the review process was initiated (registration ID: CRD42024597666).

This systematic review was conducted in accordance with the Preferred Reporting Items for Systematic Reviews and Meta-Analyses (PRISMA) guidelines [22] (see Supplementary Material Table S1 for the PRISMA checklist).

### 2.1. Search Strategy

A systematic search of the literature was conducted in three electronic databases: EMBASE, Scopus, and PubMed. The search included studies published up to 31 December 2025. The research question was formulated according to the Population, Intervention, Comparison, Outcome, and Study design (PICOS) framework [22].

1. Population: female patients with early stage breast cancer;
2. Intervention: Patient Decision Aids (PDAs);
3. Comparison: control groups (where applicable) or none;
4. Outcomes: the impact of PDAs on decision-making, decision quality, satisfaction, knowledge, usefulness, or effectiveness in treatment decision-making, as well as their acceptability, usability, and feasibility for implementation in routine clinical practice;
5. Study design: quantitative, qualitative, and mixed-methods studies.

Search terms were derived from the PICOS framework. The Boolean operators “AND” and “OR” were used to combine keywords related to breast cancer, early stage disease, and Patient Decision Aids. The final search strategy was developed in consultation with a librarian at the European Institute of Oncology. It was initially designed for PubMed and subsequently adapted for Scopus and EMBASE by translating the appropriate MeSH terms and controlled vocabulary into the corresponding database-specific indexing systems. The complete search strategies for all three databases are reported in Supplementary Material Table S2.

### 2.2. Eligibility Criteria

This review was designed to provide a focused synthesis of the evidence on Patient Decision Aids (PDAs) for women with early stage breast cancer within the SDM framework. Consequently, the eligibility criteria were intentionally defined to maximize the clinical and conceptual homogeneity of the included studies, while acknowledging that this approach excluded broader decision-support interventions and populations outside the prespecified scope.

All study designs were considered eligible, including quantitative, qualitative, and mixed-methods studies. Studies were included if they met the following eligibility criteria: (i) were published in English in peer-reviewed scientific journals; (ii) included women with early stage breast cancer, defined according to the American Joint Committee on Cancer (AJCC) staging system as non-metastatic stages 0–III [3]; (iii) investigated the impact of one or more PDAs on decision-making, regardless of the treatment option addressed, and/or evaluated the acceptability, feasibility, usability, or comprehensibility of one or more PDAs; and (iv) explicitly identified the intervention as a Patient Decision Aid developed to support SDM, thereby ensuring conceptual consistency with the theoretical framework of the review.

To ensure a clinically homogeneous study population, this review was intentionally restricted to women with early stage breast cancer. When studies including both early- and later-stage breast cancer populations were identified, they were included only if the study population consisted predominantly of women with early stage disease (AJCC stages 0–III, non-metastatic).

Studies exploring the use of PDAs among women with lower socioeconomic status (SES) or limited health literacy were also eligible for inclusion.

Study protocols, conference abstracts, and conference posters were excluded. Studies evaluating multicomponent interventions were also excluded if the independent effects of the Patient Decision Aid could not be distinguished from those of the other intervention components, as the primary objective of this review was to evaluate the effectiveness of PDAs.

No restrictions were applied regarding the year of publication. The search was conducted from database inception to 31 December 2025.

### 2.3. Selection of Sources of Evidence

All records identified through the search strategy were imported into Rayyan [23] for screening and study selection. After duplicate records were removed, the remaining articles were arranged alphabetically by the first author’s surname. Three independent reviewers (SS, BC, and LC) assessed study eligibility. First, titles and abstracts were independently screened and classified as “included,” “excluded,” or “maybe.” During this stage, the blind mode available in Rayyan was enabled to ensure independent assessments by all reviewers. The blind mode was subsequently disabled to identify agreements and discrepancies between reviewers. Any disagreements were resolved through discussion and, when necessary, by consulting a fourth independent reviewer (RG).

Studies classified as “included” were then retrieved and independently assessed in full text by the three reviewers (SS, BC, and LC). Articles for which eligibility remained uncertain were discussed among the reviewers (SS, BC, LC, and RG) until consensus was reached.

### 2.4. Data Extraction Process

All data were extracted by one reviewer (SS) and independently verified for accuracy and completeness by two additional reviewers (BC and LC) using the same Microsoft Excel spreadsheet (Microsoft Excel 2016, Microsoft Corporation). The extracted data included: study characteristics (authors, year of publication, and country in which the study was conducted); participant characteristics (sample size, age, educational level, and breast cancer stage); characteristics of the Patient Decision Aid (type, format, and content); study design; outcomes of interest; and the main findings.

### 2.5. Quality Assessment of the Included Studies

The methodological quality of the included studies was assessed using the Mixed Methods Appraisal Tool (MMAT) [24]. Three reviewers (SS, BC, and LC) independently conducted the quality assessment. The MMAT is designed to appraise five categories of study designs: qualitative, quantitative randomized controlled, quantitative non-randomized, quantitative descriptive, and mixed-methods studies. The tool begins with two screening questions applicable to all study designs: “Are there clear research questions?” and “Do the collected data allow the research questions to be addressed?” These are followed by five criteria specific to each study design category. Each criterion is rated as “Yes,” “No,” or “Cannot tell,” with the latter indicating that insufficient information was available to determine whether the criterion had been met.

Any disagreements between reviewers were resolved through discussion and, when necessary, by consultation with two independent reviewers (VS and RG). Study quality was subsequently classified as low (MMAT score 2–4), moderate (MMAT scores 5–6), or high (MMAT score 7).

### 2.6. Data Synthesis and Analysis

Due to the methodological heterogeneity of the included studies, including differences in study design (quantitative, qualitative, and mixed-methods), PDA characteristics, outcome measures, and assessment tools, statistical pooling of the results was not feasible. A narrative synthesis was therefore conducted. Studies were summarized and compared according to their study design, characteristics of the patient decision aids, and the outcomes investigated. Findings were synthesized descriptively, highlighting similarities and differences across studies regarding the effectiveness of PDAs on decision-related outcomes, patient knowledge, patient psychological well-being, patient satisfaction, acceptability, usability, and feasibility of implementation. The narrative synthesis was initially conducted by one reviewer (SS) and subsequently discussed and refined with the other reviewers (LC, BC) to ensure consistency in the interpretation of the findings.

## 3. Results

**Figure 1:**
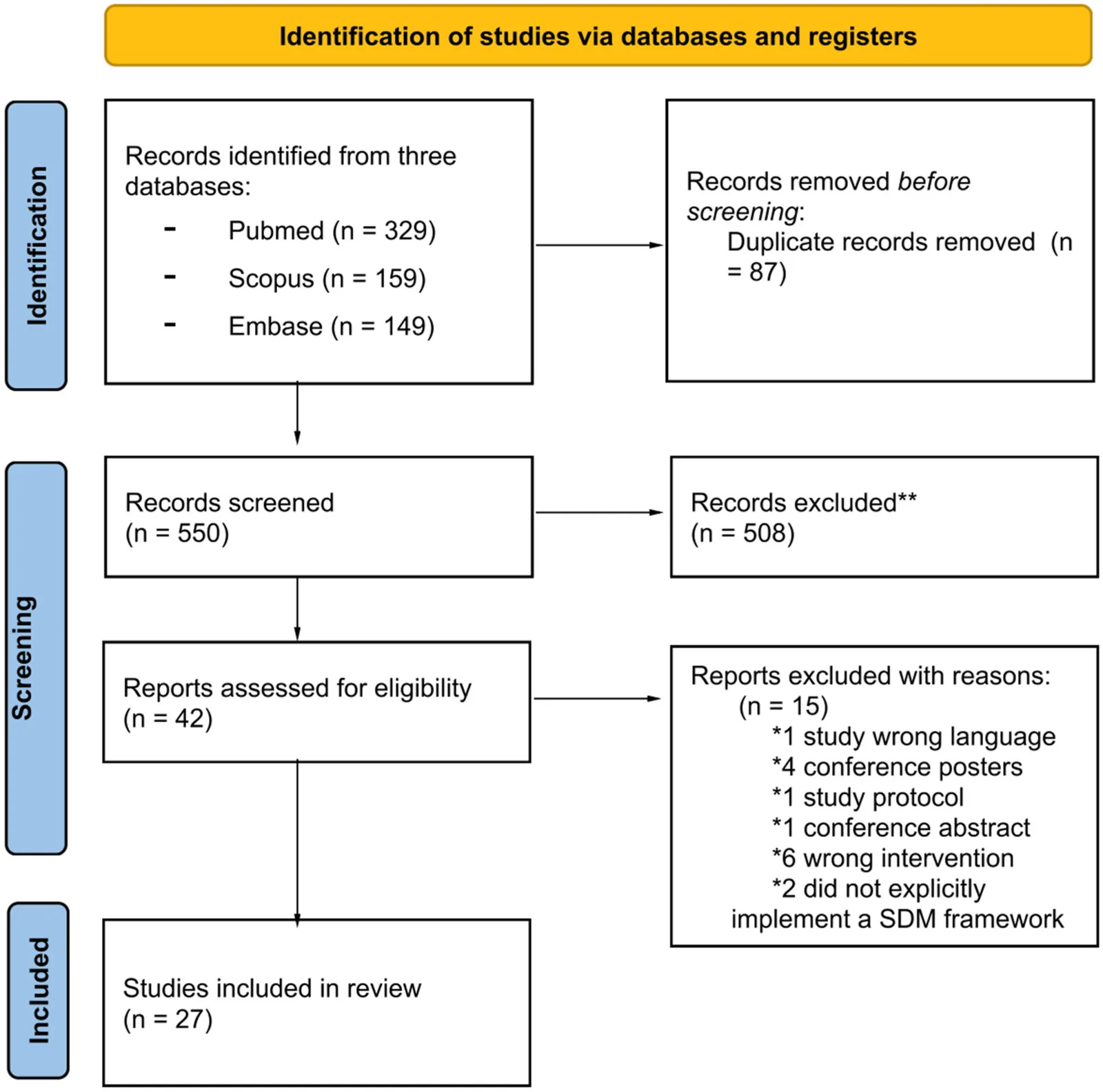
PRISMA flow diagram for study selection. The symbol (*) indicates studies excluded with reason.

A total of 26 studies were included in this review. The study selection process is illustrated in the PRISMA flow diagram presented in Figure 1. Table 1 shows sociodemographic characteristics of the included studies, as well as the type, format and content of PDA implemented. Although most included studies evaluated multiple outcomes, often using both quantitative and qualitative methodologies, several outcome domains emerged consistently across the literature. Organizing the Results by intervention type or by individual study would therefore have resulted in substantial overlap and repetition. To improve the clarity and readability of the findings, the evidence was synthesized according to the principal outcome domains. Specifically, quantitative findings are presented according to their primary focus (decision-related processes, patient knowledge, and psychological well-being), followed by qualitative findings on PDA acceptability and usability, evidence relating to patients with lower socioeconomic status or limited health literacy, and, finally, implementation issues identified across the included studies. Accordingly, Table 2 shows assessed variables and main findings, as well as study design and total MMAT score, to maximize the readability of the results.

### 3.1. Included Studies Characteristics

#### 3.1.1. Sociodemographic Characteristics of the Included Studies

This systematic review included 26 studies conducted across three continents—Europe, Asia, and North America—with the majority of studies carried out in the United States (*n* = 19), followed by Canada (*n* = 3), the Netherlands (*n* = 2), China (*n* = 1), Japan (*n* = 1), and India (*n* = 1). The studies were published between 1998 and 2025. Sample sizes ranged from 12 to 616 participants, for a total of 5174 participants, whose ages ranged from 23 to 84 years. Participants’ educational level was reported in 23 of the 26 included studies and is summarized in Table 1.

All included studies exclusively enrolled women with early stage breast cancer or study populations predominantly composed of patients with non-metastatic AJCC stages 0–III disease. Only one study including a mixed-stage population was eligible for inclusion [32], as 85% of its participants had early stage breast cancer, whereas 15% had stage III or IV disease.

#### 3.1.2. Characteristics of PDAs Implemented by the Included Studies

All included studies evaluated at least one Patient Decision Aid (PDA), delivered either in paper-based or web-based format. Specifically, 13 studies evaluated paper-based PDAs, whereas the remaining 13 implemented web-based PDAs, including text- and image-based platforms, videos, or interactive multimedia modules. One study additionally incorporated an audiotape to complement a paper-based workbook.

Four studies [26,27,35,37] implemented the Picture Option Grid [52] as a paper-based PDA. One of these studies [37] also evaluated the Comic Option Grid, an adaptation of the Picture Option Grid specifically developed for individuals with lower socioeconomic status (SES) and limited health literacy. Another study included in this review [41] evaluated a version of the Option Grid adapted for underserved populations. Three studies [33,45,46] used a video-based PDA designed as a soap opera-style narrative depicting the journey of a woman with early stage breast cancer, from diagnosis through treatment discussion, decision-making, and completion of treatment. The remaining 17 studies evaluated PDAs specifically developed to address the objectives of each individual study.

Although the format of the PDAs varied, their content was largely comparable across studies. The most frequently covered topics included frequently asked questions, information on surgical treatment options, general information about breast cancer, endocrine therapy, chemotherapy, radiotherapy, and treatment-related adverse effects. In 19 of the 26 included studies, PDAs supported decisions regarding breast-conserving surgery with radiotherapy, mastectomy, and breast reconstruction. The remaining seven studies evaluated PDAs focusing on patient narratives and lived experiences, providing general information about breast cancer and supporting decisions regarding adjuvant or neoadjuvant treatments, including endocrine therapy, immunotherapy, chemotherapy, and radiotherapy.

#### 3.1.3. Quality Assessment of Included Studies

Study quality was classified according to MMAT total scores as low (2–4), moderate (5–6), or high (7). Of the 26 included studies, five were rated as low quality, 12 as moderate quality, and nine as high quality. The most consistent benefits of PDAs—particularly reductions in decisional conflict and improvements in satisfaction with decision-making—were observed across studies of moderate and high methodological quality. In contrast, some less consistent outcomes (e.g., knowledge, psychological well-being) were reported by a smaller number of studies and, in some cases, by studies with lower MMAT ratings. These patterns suggest that confidence in the main conclusions is greater for decision-related outcomes than for psychological or quality-of-life outcomes. Full quality assessment is available in Appendix A, Table A1.

### 3.2. Quantitative Results

#### 3.2.1. Decision-Related Outcomes

Twelve studies evaluated decision-related outcomes, including decisional conflict, satisfaction with the treatment decision, satisfaction with the decision-making experience, decision quality, decisional regret, decision uncertainty, and perceived decisional support.

Seven studies [25,29,32,36,39,42,50] assessed the effect of PDAs on decisional conflict. Six of the seven studies reported that PDAs significantly reduced decisional conflict, whereas Politi et al. (2024) found no significant differences between the intervention and control groups.

Four studies examined satisfaction, although they evaluated different aspects of this construct. Two studies reported greater satisfaction with the final treatment decision among participants who used a PDA [28,40], while three others found higher satisfaction with the decision-making experience—that is, patients’ overall experience of participating in the treatment decision [39,42,44]. In contrast, one study [43] reported no effect of the PDA on overall satisfaction.

Two studies evaluated the quality of the decision-making process, referring to the extent to which patients were informed and actively involved in treatment deliberation [43,44]. Molenaar et al. (2001) reported improvements following PDA implementation, whereas Wilkins et al. (2006) found no significant differences between the intervention and control groups [43,44].

Additional decision-related outcomes were reported less frequently. Lam et al. (2013) found lower levels of decisional regret among participants who received a PDA compared with controls [29]. Joshi et al. (2023) reported reduced decision uncertainty in the intervention group, while Marziliano et al. (2023) observed significantly greater perceived decisional support among PDA users than non-users (*p* = 0.04) [25,30].

#### 3.2.2. Knowledge-Related Outcomes

Eight of the included studies explored the effect of a PDA on patients’ knowledge regarding breast cancer and possible treatments and side effects. Four out of these eight studies found that a PDA intervention has a statistically significant effect on patients’ medical knowledge [31,32,42,48], with two other studies finding a slight increase in participants’ knowledge levels, although not statistically significant [28,50]. On the other hand, two out of the eight studies that investigated this variable found that PDA implementation had no effect on patients’ medical knowledge levels with respect to participants who were not exposed to the PDA [29,43].

#### 3.2.3. Psychological Well-Being and Quality of Life

Five of the included studies investigated the impact of PDA implementation on patients’ psychological well-being and quality of life. More specifically, three studies [29,39,42] investigated both anxiety and depression scores. None of these found a significant difference in anxiety scores between patients who received the PDA intervention and those in the control groups. However, while Whelan and collaborators (2004) found no differences in depression scores between patients who had been exposed to the PDA and participants who had not, studies conducted by Osaka & Nakayama and colleagues (2017) and by Lam and collaborators (2013) found decreased depression scores among populations who had been part of the PDA intervention. Marziliano and collaborators (2023), while evaluating the impact of a PDA, measured psychological distress, finding that more patients in the intervention arm met the criteria for clinically significant psychological distress compared with patients in the control arm (*p* = 0.05) [30]. Molenaar and colleagues (2001) aimed to investigate the effects of an online PDA on quality of life, observing an overall positive effect of the PDA (*p* < 0.01) (*p* = 0.05) [44].

### 3.3. Qualitative Outcomes Related to PDAs Implemented

Nine of the included studies qualitatively evaluated the implementation of a PDA for early stage BC patients [25,26,31,34,36,38,40,43,49]. All participants positively reviewed the DA and reported it as being helpful and useful in facilitating SDM with their physicians. The majority of these studies also reported the PDA implementation as being highly feasible and acceptable; however, some concerns emerged. For instance, in the study conducted by Pesavento and colleagues (2024), patients reported that different recommendations from different surgeons made it difficult to interpret the PDA’s suggestions. Participants who took part in the study conducted by Sawka and colleagues (2002) also reported that their emotional state at diagnosis hindered information absorption. The study from Schonberg and collaborators (2019) highlights the preference among older women for a paper-based instead of a web-based PDA. Finally, qualitative findings from D’Alimonte and collaborators (2012) and from Sawka and colleagues’ work (2002) highlighted some implementation issues that will be further discussed in the dedicated Section 3.5—Other outcomes related to PDAs implementation.

### 3.4. Outcomes from Lower SES and Lower Literacy Populations

Ten of the included studies focused on the implementation of a PDA for lower SES and lower literacy populations [26,27,33,35,37,41,45,46,47,51]. Three of these highlighted the importance of pictures within a PDA specifically aimed at lower literacy populations, due to pictorial superiority, with Durand and collaborators (2021) finding higher knowledge scores (*p* = 0.04), higher SDM scores (*p* = 0.01), higher decision process subscale scores (*p* = 0.05), and lower decision regret at follow-up (*p* = 0.04) with respect to the control group when introducing a pictorial PDA. Lower decisional regret was also found among lower SES and lower literacy populations in the studies conducted by Jibaja and colleagues (2011, 2006). Qualitative analyses performed on lower SES and lower literacy populations show participants favorably reviewing the PDA, reporting a decrease in their worries and higher medical knowledge. Stankowski-Drengler and collaborators (2019) noted a difference in satisfaction with the decision process by education level: more educated patients reported higher satisfaction with the decision process. Nonetheless, Yen and collaborators (2020) found that lower SES patients reported higher SDM scores in the control arm, who were not subjected to PDA exposure, while Schumacher and colleagues (2025) found no statistically significant difference in the impact of the PDA on active patients’ behavior based on socioeconomic disadvantage and that socioeconomic disadvantage was, in fact, associated with significantly lower levels of engagement (*p* = 0.006).

### 3.5. Other Outcomes Related to PDA Implementation

Six studies out of the 26 included in the present work also highlighted noteworthy outcomes related to various implementation issues or suggestions coming directly from participants. For instance, Schubbe and collaborators (2021), while implementing a PDA within their standard oncological consultations, noted that physicians needed a brief training period before the PDA could be flawlessly implemented within their consultations without interfering with their schedules [26]. Participants in the study conducted by Schonberg and colleagues (2019) reported preferring the PDA to be given to them ahead of their first oncological consultation, while patients included in the study by D’Alimonte and collaborators (2012) also noted the importance of being promptly given access to the PDA, with older patients also suggesting that a supporting figure (such as a medical provider or peer helper) be made available to support them while going through the tool. Minami and colleagues (2021) found that different patients have different preferences regarding their involvement in decision-making and when to receive the PDA [31,38,49].

Molenaar and collaborators (2001) implemented the same PDA in three different hospitals with experimental groups and wait-list control groups, and found a significant effect of the variable “hospital” on satisfaction (F(18;270) = 1.80; *p* = 0.03) [44]. Finally, Stankowski-Drengler and collaborators (2019) found that patient perception of being asked about their preference varied by surgeon seen (*p* < 0.0001) and that the surgeon seen was more important than randomization arm or patient factors in predicting patients’ perception of information conveyed during the surgical consultation [47].

## 4. Discussion

The present work aimed at gathering information on already available PDA for early stage BC patients implemented within an SDM framework. The inclusion of both qualitative and quantitative measures provided a clear overview of current interventions, the main variables used to assess PDAs’efficacy, and qualitative feedback on available DAs coming straight from early stage BC patients, while also making it possible to collect information from different socio-economic strata.

Outcomes from quantitative studies show that the use of a PDA has a positive impact on decision quality, satisfaction with the decision and with the decision-making process itself, while also reducing decision conflict and decision regret. This is in line with most recent systematic reviews [11]; however, the literature also reports inconclusive or contrasting results regarding the efficacy of PDAs in reducing decisional conflict [53]. A statistically significant increase in knowledge regarding BC or its treatment has been found in four out of the eight studies that assessed this variable, with two more studies finding a not significant increase. A positive increase in knowledge level is widely recognized across most recent reviews [54]. However, our findings’ inconsistency could be due to methodological issues of the included studies or to the fact that knowledge relates more to the subjective characteristic of the patient rather than the PDA per se: for instance, one could have more or less previous medical knowledge than another, as found by Belkora and collaborators (2012) and as previously reported across the literature [32,55]. The majority of the included studies that focused on patients’ psychological well-being showed no significant changes in depression or anxiety levels were observed with the implementation of a PDA; while QoL levels seem to increase, together with general psychological distress, in a minority of studies. However, given the mental, physical and psychological load that oncological patients deal with on a daily basis [56], it is difficult to discern how much psychological distress is caused by the implementation of a DA and how much is related to contextual issues regarding the decision-making itself, which usually turns out to be impacted by anxiety and mental health dysfunctions [57,58]. Given these challenges, future PDA developers may want to focus their research on providing psychological interventions to teach BC patients to better regulate their emotions [59].

Outcomes related to implementation issues provide us with important supplemental information regarding the enactment of a DA within standard clinical practices. First and foremost, data emerged from Minami and collaborators’ work (2021) indicate that not all patients may want to be involved in treatment decision-making at the same level [31]. It is therefore crucial to assess the preferred level of participation of each patient, in order to tailor the tool specifically to their needs, as previously also reported across the literature [60,61]. Furthermore, emerging data from two other of the included studies [44,47] call attention to the possibility that the healthcare professional seen or the hospital in which the intervention takes place could have a bigger effect on the efficacy of the DAs than the tools themselves. This could imply the existence of systemic differences across healthcare professionals’ ways of conducting consultations or differences in policy implementation across hospitals, as reported by the recent literature [62,63]. These issues, if not properly addressed, could limit the effectiveness of PDAs, with patients deriving less benefit from their implementation. Although the initial introduction of PDAs may entail short-term costs and additional time for training and workflow adaptation, the broader literature on personalized and shared decision-making interventions suggests that such approaches have the potential to improve care efficiency and reduce downstream resource use over time [64,65]. However, none of the studies included in this review directly assessed costs or cost-effectiveness, and therefore no firm conclusions can be drawn regarding the economic impact of PDAs in early stage breast cancer care.

Findings from qualitative outcomes show that all BC patients would recommend the use of a PDA to other patients. This is of great importance, as the literature shows the role of peers as being one of the most crucial facilitators towards patients’ empowerment [66] and peer recommendations could therefore improve all benefits brought about by a DA implementation.

Finally, findings regarding lower socio-economic status and/or lower health literacy populations showed that in the majority of the included studies, the use of a PDA improves SDM. However, not all of the included studies report this positive effect of a DA on SDM or the decision-making process. This is in line with results from the literature that highlights the contrasting results of a DA implementation in decreasing inequity in health care, also pointing to further research being needed to address undeserving populations’ needs [67]. Furthermore, our findings point to the use of pictures and images in a PDA being a powerful tool to increase both the quantity of information conveyed and the clarity of said information. This is in line with the commonly recognized concept of pictorial superiority, according to which it is much easier to remember pictures than words, as it requires less cognitive effort. Since people with lower literacy and/or from a lower socio-economic status usually lack the tools to correctly identify and understand labels and health messages [68,69], using pictorial superiority in treatment decision aids might be useful to shorten this knowledge gap across socioeconomic strata. This is also supported by qualitative outcomes that emerged from the present work that focused on lower-literacy women: plain language and images seem to guarantee a more feasible and acceptable PDA.

An additional consideration is that the included studies span more than two decades, during which both PDA design and shared decision-making practices have evolved substantially. When studies were considered chronologically, no clear linear improvement in PDA effectiveness was apparent. Positive findings were reported in both earlier and more recent studies, while null findings were also present among newer interventions. More recent studies tended to use web-based or interactive formats and more frequently examined implementation and SDM, but the heterogeneity of outcomes and the small number of studies within each period prevented a formal assessment of temporal effects. Among the studies that directly assessed SDM, positive findings were observed in some interventions [35,41], whereas other findings [47,50] reported no significant improvement in SDM-related outcomes. This pattern is compatible with the possibility that PDA effects depend on the quality of the patient–clinician interaction; however, the included studies used different measures, and SDM scores were not consistently reported. Consequently, a relationship between higher SDM levels and better outcomes could not be formally examined.

The included studies did not provide sufficient information to classify all PDAs according to IPDAS guidelines [70] or other established PDA development criteria. Therefore, a direct comparison between well-developed and moderately developed PDAs was not possible. Nevertheless, the available descriptions suggest considerable variation in the development process, including differences in patient involvement, values clarification, clinician feedback and usability testing. This variation may have contributed to the heterogeneous results, particularly because the most consistent positive findings were not restricted to a single PDA format or development model.

The observed pattern suggests that the most consistent evidence concerns decision-related outcomes, particularly decisional conflict, which were reported across studies with different MMAT ratings but were also supported by several moderate- and high-quality studies. This reduces the likelihood that the finding is attributable solely to low methodological quality, although the presence of low-quality studies and the absence of quantitative pooling limit the strength of this inference.

Overall, in women with early stage breast cancer, PDAs appear to support knowledge and may improve selected decision-related outcomes. However, evidence for psychological outcomes and quality of life is inconsistent, and not all studies show benefit.

Given the heterogenicity of assessed variables and measurement tools highlighted by the present work, it remains difficult to discern which PDA features or implementation strategies are most helpful for early stage breast cancer patients facing treatment decisions. Furthermore, as also highlighted in previous recent works [71], many PDAs are constantly being developed without meeting the IPDAS [70] criteria for standardized PDA development. Future research should therefore focus on higher-quality study designs, standardized outcome measurement, and better integration of PDAs into the clinical decision-making process.

## 5. Strengths and Limitations

This review has several strengths. First, it was conducted in accordance with PRISMA guidelines and based on a prospectively registered protocol (PROSPERO CRD42024597666), which enhances transparency and reduces the risk of selective reporting. The search strategy was comprehensive, covering three major databases (PubMed, EMBASE, and Scopus) from inception to December 2025, and was developed in consultation with an experienced librarian to ensure appropriate use of controlled vocabulary and search terms. Study selection, data extraction, and quality appraisal were performed independently by multiple reviewers, with disagreements resolved through discussion and, when necessary, consultation with additional reviewers, thereby minimizing bias and improving the reliability of the included evidence.

Second, the review provides a broad and clinically relevant synthesis of the evidence on PDAs for women with early stage breast cancer within a shared decision-making framework. By including quantitative, qualitative, and mixed-methods studies, the review captures both effectiveness and implementation-related insights, offering a more complete picture of how PDAs function in real-world settings. The explicit focus on underserved populations, including women with lower socioeconomic status and limited health literacy, further strengthens the clinical and policy relevance of the findings, as it highlights which PDA formats and features may help reduce inequities in decision-making support. Finally, the use of the MMAT allowed for a structured appraisal of methodological quality across diverse study designs, enabling a more nuanced interpretation of the consistency and robustness of the reported effects.

Several limitations should be acknowledged. First, the included studies were characterized by substantial heterogeneity in design, populations, and outcome measures, which limits the generalizability of the findings to the broader population of women with early stage breast cancer. For example, instruments used to assess knowledge were typically developed ad hoc for each specific PDA and study, and most lacked external validation or psychometric testing. This variability reduces the comparability of results across studies and may affect the reliability of some outcomes. In addition, the construct of “decision and decision-making” was operationalized in different ways across studies (e.g., decisional conflict, satisfaction with the decision, decision quality, decisional regret), making it difficult to draw a unified and definitive conclusion about the overall impact of PDAs on decision-related outcomes.

Second, this review focused exclusively on treatment decision-making at the time of initial diagnosis and did not capture the longitudinal and multifaceted nature of decision-making in breast cancer care. As O’Brien and colleagues have noted [72] treatment decision-making for oncological patients is an ongoing process in which women are required to make multiple, sequential decisions that can profoundly affect their lives, self-perception, and quality of life. Following the initial treatment choice, patients may face additional decisions regarding breast reconstruction, preventive bilateral mastectomy, adjuvant therapies, surveillance strategies, and other preventive measures aimed at reducing recurrence risk or restoring a sense of control over their bodies. The present review was not designed to evaluate these later decision-making phases; its objective was specifically to synthesize evidence on PDAs supporting treatment decision-making in women newly diagnosed with early stage breast cancer. Consequently, the findings do not reflect the full trajectory of decision support needs across the cancer care continuum.

A final limitation is that this review examined PDAs as stand-alone interventions, whereas in clinical practice they are often implemented as part of broader, multicomponent strategies [54]. Evidence from implementation research suggests that such combined approaches may be more effective than stand-alone tools [73] but studies evaluating multicomponent interventions were largely excluded or could not be disentangled in the present synthesis. Consequently, the findings may underestimate the potential impact of PDAs when implemented together with comprehensive shared decision-making interventions.

## 6. Conclusions and Clinical Implications

This systematic review provides a comprehensive overview of the current evidence on PDAs for women with early stage breast cancer. Overall, PDAs were associated with improved decision quality, greater satisfaction with the decision-making process, and, in some studies, enhanced knowledge about treatment options, without consistent increases in anxiety or depression. However, improvements in knowledge, psychological outcomes, quality of life, and decision-related outcomes were not uniform across all studies or outcomes: some reported statistically significant benefits, whereas others found no effect or only non-significant trends. The most consistent and robust findings—particularly reductions in decisional conflict and improvements in satisfaction—were generally supported by studies rated as moderate or high quality using the MMAT, whereas evidence for psychological and quality-of-life outcomes was more heterogeneous and often based on fewer or lower-quality studies. Tailored approaches, especially those employing pictorial or simplified formats, appeared effective in improving understanding and decision-making among patients with lower health literacy or from disadvantaged socioeconomic backgrounds. Nevertheless, the heterogeneity of study designs, the variability in outcome measures, the limited external validation of assessment tools, and the variable methodological quality constrain the generalizability and certainty of these findings. Contextual factors, such as prior knowledge, psychological distress, and the broader, ongoing nature of treatment-related decisions, may further influence the observed effects of PDAs.

The findings of this review highlight the potential of PDAs as practical tools to support SDM in early stage breast cancer care, while underscoring the need for cautious interpretation of the evidence. Clinicians should consider each patient’s preferred level of participation and adapt PDAs to individual literacy, cognitive load, and socioeconomic circumstances. The integration of visual aids, plain language, and structured content can reduce cognitive barriers, enhance comprehension, and mitigate disparities in healthcare access and understanding. Successful implementation of PDAs also requires appropriate institutional policies, organizational support, and careful workflow integration to avoid disruption during consultations. While short-term costs and time investments may be perceived as barriers, evidence suggests that well-implemented PDAs can contribute to more efficient, patient-centered, and potentially cost-effective care in the long term. Peer recommendation and patient endorsement further enhance PDA acceptability, supporting their broader adoption in oncology practice. Clinicians and healthcare systems should, therefore, prioritize the development, customization, and structured implementation of PDAs to maximize their impact on informed and equitable decision-making for women facing early stage breast cancer treatment choices, while recognizing that the current evidence does not support a uniform beneficial effect across all outcomes and that higher-quality, standardized research is needed to clarify which PDA features and implementation strategies yield the greatest benefit.

## Figures and Tables

**Table 1:** Sociodemographic characteristics of the included studies: name, format and content of PDAs implemented.

STUDY	COUNTRY	SAMPLE SIZE	AGE (Years)	LEVEL OF EDUCATION; HEALTH LITERACY; SES	BREAST TUMOR STAGES	Type of PDA and FORMAT	PDA Content
Joshi, S. et al., 2023 [25]	India	N = 245;	median 48; range 23–76	58,6% lower or middle SES, 46.4% did not complete college	median tumor size 2.5 cm (0–6);	Navya Patient Preference Tool (Navya-PPT)—online-based	Surgical options; cosmetic outcomes; radiation; treatment costs; patient preferences.
[26]	USA	N = 43	mean 56.6; SD 12.0	26%lower SES; 2% never attended high school; 26% high school diploma; 21% some college; 14% 2-year degree; 37% 4-year degree or higher	I–IIIA stages	Option Grid; Picture Option Grid—paper-based	Surgical options; FAQs; survival; recurrence; chemotherapy information.
[27]	USA	N = 616;	mean 59.7; SD 12.5	44.8% low health literacy; 33% lower SES	I–IIIA stages	Option Grid; Picture Option Grid—paper-based	Surgical options; FAQs; survival; recurrence; chemotherapy information.
[28]	USA	N = 101	mean 53; range 30–80	66% some college or more	0–II stages	unique to the study—online-based	Surgical options; risks/benefits; values clarification; communication support; recurrence and cosmetic outcomes.
[29]	China	N = 276	Experimental group: mean 56.8; SD 10.8; CG: mean 54.6; SD 10.1	EG: 10.9% tertiary education; 51.4% secondary education; 37.7% primary or no formal education. CG: 10.1% tertiary educaton; 52.9% secondary education; 37% primary or no formal education	I–III stages	unique to the study—paper-based	Treatment options; pros/cons; values clarification; decision guidance.
[30]	USA	N = 388	mean 55.50; SD 11.13	48.7% completed college or higher	non metastatic	Healing Choices—online, interactive	Educational resources; patient stories; question prompts; information management.
[31]	USA	N = 33	mean 74.4; SD 3.8	72% college graduates	I stage	unique to the study—paper-based	Surgical, radiation and endocrine treatment information.
[32]	USA	N = 437	mean 54; median 54	32% lower than college graduates; 32% college graduates; 34% higher than college graduates	18% stage 0; 67% stage I or II; 15% stage III or IV	5 different PDAs unique to the study—online-based	Five PDAs covering surgery, reconstruction, adjuvant therapy, DCIS and metastatic disease.
[33]	USA	N = 76	EG: mean 49.5; SD 10.3. CG: mean 52.4; SD 12.1	patients have no medical insurance and many have a high school education or less	I–IIIA stages	unique to the study—online, interactive	Interactive patient story; treatment information; decision guidance; common concerns.
[34]	Netherlands	N = 60 [N = 40 quantitative data; N = 20 qualitative data]	Quantitative data: mean 54, SD 10.1 Qualitative data: mean 53; SD 9.7	N = 4 primary and lower secondary education; N = 7 upper secondary education, post-secondary education and short cycle tertiary education; N = 9 bachelor, master or doctorate degrees	I or II stages	unique to the study—online-based	Surgical information; SDM implementation; communication support.
[35]	USA	N = 311	mean 60.5; SD 12.2	education received = 64.6% less than college degree; 35.4% 4-year college degree or higher. health literacy = 54% adequate, 45.3% inadequate, 0.6% missing/prefer not to say. 66.6% higher SES	I–IIIA stages	Option Grid; Picture Option Grid—paper-based	Surgical options; FAQs; survival; recurrence; chemotherapy information.
[36]	Canada	N = 50 [Focus group N = 22; Pilot 1: N = 18; Pilot 2 N = 10]	Pilot 1: mean 55; median 52; range 37–74. Pilot 2: mean 52; median 43; range 34–78	Pilot 1: N = 5 high school; N = 6 some college; N = 7 some university. Pilot 2: N = 3 high school, N = 4 some college; N = 3 some university	I or II stages	unique to the study—paper-based + audio	Surgery; radiation; recurrence; complications; reconstruction; values clarification.
[37]	USA	N = 288 [N = 5 focus group; N = 268 web-based questionnaire; N = 15 Interviews]	Focus group: N/A Web-based questionnaires: 79% between 45 and 74 years Interviews: 40% between 55 and 64 years; mean 56.8; SE 4.40	Focus group: N/A Web-based questionnaires: 1.5% some high school or less; 22.8% high school graduate; 27.2% some college/technical school; 24.3% college graduate. Interviews: N = 10 women of lower SES	I to IIIC stages	Option Grid; Picture Option Grid; Comic Option Grid (adapted for a low SES and low literacy populations)—paper-based	Surgical options; FAQs; survival; recurrence; chemotherapy information.
[38]	USA	N = 35	mean 74.3; SD 3.3	6% high school; 26% some college; 29% completed college; 40% beyond college	early stage, max 3 cm	unique to the study—paper-based	General health; treatment options; values clarification; endocrine therapy; FAQs.
[39]	Japan	N = 210	DA + Narratives EG: mean 50.2; SD 10.7 DA EG: mean 49.7; SD 9.9. CG: mean 48.6; SD 8.9.	DA + Narratives EG: 25.9% high school; 44.8% technical school; 27.6% college; 1.7%graduate. DA EG: 21.3% High school; 50.9% technical school; 26.2% college; 1.6% graduate. CG: 18.2% High school; 38.2% technical school; 36.4% college; 7.3% graduate.	0–III stage	developed ad hoc; developed ad hoc + patients narratives—paper-based	Surgical options; benefits/harms; values clarification; decision guidance; patient narratives.
[40]	USA	N = 16	median 75; range 72–79	12,5% high school or less; 12,5% some college; 25% bachelor; 50% higher education	early stage, noninvasive	unique to the study—paper-based	Treatment options; overall health; endocrine therapy; values clarification; FAQs.
[41]	USA	N = 81 [N = 18 & N = 53 for phase 1; N = 10 for phase 2]	mean 56.8; range 31–75	N = 7 high school or less; N = 2 some college; N = 1 graduate	I to IIIA stages	Option Grid Adapted—paper-based	Surgical options; FAQs; survival; recurrence; chemotherapy information.
[42]	Canada	N = 201	EG: median 58.2. CG: median 58.1	EG: 47% high school; CG: 50% high school	I or II stages	unique to the study—paper-based	Treatment options; adverse effects; survival; quality of life.
[43]	USA	N = 101	mean 54.9; SD 9.8	N/A	I or II stages	unique to the study—online-based	Breast cancer information; treatment risks/benefits; outcome probabilities; patient experiences.
[44]	Netherlands	N = 180	EG: mean 55.4; SD 10.8; CG: mean 54.6; SD 10.6	EG: 8% less than compulsory, 47% compusory; 21% more than compulsory, less than university; 16% university. CG: 8% less than compulsory; 51% compulsory; 14% more than compulsory, less than university; 15% university	I or II stages	CDROM—online-based	Surgical options; benefits/harms; printable consultation summary.
[45]	USA	N = 44	N/A	N/A	I–IIIB stages	unique to the study—online, interactive (soap opera)	Cancer information; misconceptions; treatment; support; personalized decision guidance.
[46]	USA	N = 44	range 29–70	N/A	I–IIIA stages	unique to the study—online, interactive (soap opera)	Cancer information; misconceptions; treatment; support; personalized decision guidance.
[47]	USA	N = 201	median full sample: 59 CG: median 58, range 27–79; EG: medan 61; range 29–80	62% college educated	0–III stages	unique to the study—online-based	Breast reconstruction; educational modules; values clarification; video vignettes.
[48]	USA	N = 227	median full sample: 59 CG: median 57, range 27–78. EG: median 61, range 29–80	65% college educated	0–III stages	unique to the study—online-based	Breast reconstruction; educational modules; values clarification; video vignettes.
[49]	Canada	N = 12	median 76, range 70–84	N/A	I stage	unique to the study—paper-based	Not reported.
[50]	USA	N = 369 [experimental group N = 184; control group N = 185]	EG: mean, 51.0, SD = 10.8; CG: 51.2, SD = 11.2	EG: High school or less 10.6% Some college 21.8% college degree 33.4% graduate 34.5%; CG: high school or less 10.1% Some college 17.1% college degree 36.7% graduate 36.1%	EG 87,6% stages 0–II; CG 88,1% stages 0–II	BREASTChoice—online-based	Breast reconstruction; complication risks; preference elicitation; clinician feedback.
[51]	USA	N = 573	median 60; range 27–90	Area Deprivation index 5 (1–10); 23% socioeconomically disadvantaged	72% of participants stage I or II	unique to the study—online-based	Not reported.

**Abbreviations:** EG = experimental group; CG = Control Group; SES = Socio-Economic Status; DA = Decision Aid; SD = Standard Deviation; N/A = Not Available.

**Table 2:** Study design, total MMAT scores and main findings from the included studies.

Study	Design	Decision-Related Processes	Knowledge	Psychological Well-Being	Acceptability/Usability	Implementation Issues	Lower SES/Health Literacy	Total MMAT Score
[25]	RCT	Improved decisional conflict; improved certainty regarding treatment preference	—	—	—	Family involvement supported PDA use	—	6
[26]	Qualitative	PDA influenced treatment decisions	—	—	PDA perceived as concise and useful	Clinicians required training before routine implementation; patients preferred receiving PDA throughout the consultation	Pictorial format preferred by lower SES patients	3
[27]	RCT	Improved decision quality; improved SDM; improved care coordination; decreased decision regret	Improved knowledge	No effect reported	—	—	Picture Option Grid reduced disparities in knowledge; SES influenced decision regret outcomes	3
[28]	RCT	Improved value concordance; improved satisfaction with decision-making	Slight improvement	—	—	—	—	3
[29]	RCT	Decreased decisional conflict; decreased decision regret; improved satisfaction with decision process	No effect	Decreased depression; no effect on anxiety	—	—	—	6
[30]	RCT	Improved perceived support during decision-making	—	Increased psychological distress	—	—	—	4
[31]	Mixed methods	Different preferences regarding involvement in SDM	Improved knowledge	—	PDA perceived positively	Timing of PDA should be individualized	—	7
[32]	Quantitative NR	Decreased decisional conflict	Improved knowledge	—	—	—	—	7
[33]	RCT	Decreased decisional conflict; improved informed choice; improved values clarity	Improved knowledge	—	Improved satisfaction	—	—	5
[34]	Qualitative	Patients perceived SDM, although preference discussions were sometimes lacking	—	—	PDA perceived as useful	Improvements needed in PDA delivery during consultations	—	7
[35]	RCT	Improved observed SDM; improved patient-reported SDM	—	—	—	—	Lower SES associated with different SDM patterns	7
[36]	Mixed methods	Decreased decisional conflict	Improved knowledge	Emotional distress hindered information uptake	Positive feedback	Home viewing suggested	—	7
[37]	Mixed methods	—	—	—	Picture Option Grid most appreciated; PDA easy to understand	Treatment costs should be addressed	Positive feedback from lower SES women	5
[38]	Qualitative	Improved preparation for decision-making	—	No effect on anxiety	Appropriate length, balanced and useful	Older women preferred receiving paper PDA before consultation	—	7
[39]	RCT	Decreased decisional conflict; improved satisfaction with decision-making	—	No effect on anxiety	—	—	—	5
[40]	Quantitative descriptive	Improved satisfaction with decision	—	—	High acceptability and usability	Different surgeon recommendations complicated implementation	—	6
[41]	Qualitative	—	—	—	PDA considered feasible, useful and acceptable	Pictorial format appreciated	Better accessibility for patients with limited literacy	6
[42]	RCT	Decreased decisional conflict; improved satisfaction with decision-making	Improved knowledge	No effect on anxiety or depression	—	—	—	6
[43]	Quantitative NR	No effect	No effect	No effect	PDA positively evaluated	—	—	7
[44]	Quantitative NR	Improved satisfaction with decision-making	—	Improved quality of life	—	—	—	7
[45]	Quantitative NR	—	Improved knowledge	—	PDA positively evaluated	—	—	6
[46]	Quantitative NR	Decreased decisional conflict; improved clarity about treatment options	—	—	—	—	—	6
[47]	RCT	No effect of PDA on perceived SDM; surgeon influence predominated	—	—	—	Surgeon-related factors influenced implementation	Education level influenced satisfaction	4
[48]	RCT	—	Improved knowledge	—	PDA perceived as useful	—	—	6
[49]	Qualitative	—	—	—	PDA considered acceptable, informative and visually appealing	Timing and support barriers identified	—	7
[50]	RCT	No effect on decisional conflict or SDM	No significant improvement	—	Good usability	—	—	6
[51]	RCT	No effect on patient engagement	—	—	—	—	Socioeconomic disadvantage associated with lower engagement	5

## Data Availability

No new data were created or analyzed in this study. Data sharing is not applicable to this article.

## Supplementary Materials

The following supporting information can be downloaded at: https://www.mdpi.com/article/10.3390/curroncol33090518/s1, Table S1: PRISMA checklists for systematic reviews; Table S2: Search strategies.

## Appendix A

**Table A1:** Quality assessment of the included studies according to MMAT.

STUDY	SCREENING QUESTIONS	QUANTITATIVE STUDIES	QUALITATIVE STUDIES	MIXED METHODS STUDIES	TOTAL MMAT SCORE
[25]	1; 1	1; 1; 1; CAN’T TELL; 1			6
[26]	0; CAN’T TELL		CAN’T TELL; CAN’T TELL; 1; 1; 1		3
[27]	1; 1	CAN’T TELL; 0; 0; CAN’T TELL; 1			3
[28]	1; 1	0; CAN’T TELL; CAN’T TELL; 0; 1			3
[29]	1; 1	1; 1; 1; CAN’T TELL; 1			6
[30]	1; 1	CAN’T TELL; 1; 0; 1; 0			4
[31]	1; 1			1; 1; 1; 1; 1	7
[32]	1; 1	1; 1; 1; 1; 1			7
[33]	1; 1	1; CAN’T TELL; 1; CAN’T TELL; 1			5
[34]	1; 1		1; 1; 1; 1; 1		7
[35]	1; 1	1; 1; 1; 1; 1			7
[36]	1; 1			1; 1; 1; 1; 1	7
[37]	1; 1			CAN’T TELL; 1; 1; 1; 0	5
[38]	1; 1		1; 1; 1; 1; 1		7
[39]	1; 1	1; 1; 0; CAN’T TELL; 1			5
[40]	1; 1		1; 1; 1; 0; 1		6
[41]	1; 1		0; 1; 1; 1; 1		6
[42]	1; 1	1; 1; 1; CAN’T TELL; 1			6
[43]	1; 1	1; 1; 1; 1; 1			7
[44]	1; 1	1; 1; 1; 1; 1			7
[45]	1; 1	1; 1; 1; 0; 1			6
[46]	1; 1	1; 1; 1; 0; 1			6
[47]	1; 1	1; CAN’T TELL; 0; CAN’T TELL; 1			4
[48]	1; 1	1; 1; 1; CAN’T TELL; 1			6
[49]	1; 1		1; 1; 1; 1; 1		7
[50]	1; 1	1; 1; 1; CAN’T TELL; 1			6
[51]	1; 1	1; 1; 1; CAN’T TELL; CAN’T TELL			5
